# The influence of simulator input conditions on the wear of total knee replacements: An experimental and computational study

**DOI:** 10.1177/0954411916645134

**Published:** 2016-05-09

**Authors:** Claire L Brockett, Abdellatif Abdelgaied, Tony Haythornthwaite, Catherine Hardaker, John Fisher, Louise M Jennings

**Affiliations:** 1 Institute of Medical and Biological Engineering, School of Mechanical Engineering, University of Leeds, Leeds, UK; 2 DePuy Synthes Joint Reconstruction, Leeds, UK

**Keywords:** Joint simulators, knee prostheses, orthopaedic tribology, wear analysis/testing (biomechanics), contact kinematics

## Abstract

Advancements in knee replacement design, material and sterilisation processes have provided improved clinical results. However, surface wear of the polyethylene leading to osteolysis is still considered the longer-term risk factor. Experimental wear simulation is an established method for evaluating the wear performance of total joint replacements. The aim of this study was to investigate the influence of simulation input conditions, specifically input kinematic magnitudes, waveforms and directions of motion and position of the femoral centre of rotation, on the wear performance of a fixed-bearing total knee replacement through a combined experimental and computational approach. Studies were completed using conventional and moderately cross-linked polyethylene to determine whether the influence of these simulation input conditions varied with material. The position of the femoral centre of rotation and the input kinematics were shown to have a significant influence on the wear rates. Similar trends were shown for both the conventional and moderately cross-linked polyethylene materials, although lower wear rates were found for the moderately cross-linked polyethylene due to the higher level of cross-linking. The most important factor influencing the wear was the position of the relative contact point at the femoral component and tibial insert interface. This was dependent on the combination of input displacement magnitudes, waveforms, direction of motion and femoral centre of rotation. This study provides further evidence that in order to study variables such as design and material in total knee replacement, it is important to carefully control knee simulation conditions. This can be more effectively achieved through the use of displacement control simulation.

## Introduction

Total knee replacement (TKR) is an increasingly common surgical intervention for the treatment of arthritis and joint degeneration.^1^ Advancements in implant design, material and sterilisation processes have provided improved clinical results.^2,3^ However, surface wear of the polyethylene leading to osteolysis is still considered the longer-term risk factor, particularly as life expectancy and activity levels increase.^4^

Experimental wear simulation is an established method for evaluating the wear performance of total joint replacements, with numerous publications over the last decade demonstrating the influence of design, material, size and sterilisation processes on the performance of TKRs.^5–13^ Experimental wear simulation under loading and motion representative of in vivo conditions is used to predict clinical wear performance. However, it has been shown that variation in the experimental conditions, such as kinematic inputs and component alignment, will have an impact on the wear performance of a TKR.^10,14–16^

Indeed, different research centres have adopted different approaches to experimental wear simulation of TKRs, with different conditions all too common. The resulting contact point movement that these simulations produce will depend on the combination of many parameters including input displacement magnitudes, waveforms, direction of motion and femoral centre of rotation (CoR). All of these factors will influence the relative amounts of rolling and sliding and hence polyethylene wear rate. Furthermore, the surface wear of polyethylene has been shown to be dependent on contact pressure, cross-shear and surface areas of the polyethylene being worn.^4^

The knee simulation philosophy developed in the early 2000s at the University of Leeds^10,17–19^ accounted for femoral rollback, with the femoral distal radius taken as the femoral CoR and an anterior shift of the tibia relative to femur during gait. Leeds/Prosim six-station knee simulators can be driven in either force or displacement control. The appropriate control regime is selected depending on the level of intrinsic constraint of the knee replacement. For example, displacement control being selected for prostheses that do not have intrinsic constraint within the design and rely on soft-tissue constraints in vivo. At a similar time, international standards for the wear simulation of TKRs were developed for force and displacement control regimes. The philosophy of the ISO standards appeared to be to maintain the tibia-femoral articulation within the centre of the tibial component, and therefore, a femoral CoR representing an average centre of the femoral distal and posterior radii was adopted.^20^ The ISO CoR of the femoral component combined with the ISO displacement control kinematic inputs did not, however, replicate femoral rollback.^20,21^

When comparing the displacement control profiles of the ISO standard^21^ with the Leeds profiles, both use similar waveforms and amplitudes for axial load (maximum 2600 N) and flexion–extension angle (maximum 58°). For the ISO standard, also described in the study by Johnson et al.,^22^ a maximum of 5-mm anterior–posterior (AP) displacement and internal–external (IE) rotation of between −2° and 6° was adopted.^21^ Leeds based their displacement control profiles for tibial rotation and AP translation on the data of Lafortune et al.,^23^ who analysed healthy patients without replacement prostheses. This resulted in an IE tibial rotation profile of ±5°. For AP translation, two different kinematic conditions were adopted to simulate two levels of activity, with a maximum AP displacement of either 5 mm (intermediate kinematics) or 10 mm (high kinematics).^10^

A further consideration is the direction of motion or polarities of the input kinematics. Interestingly, the maximum AP translation as defined in the displacement control standard at the time of this study^21^ was originally defined as posterior tibia translation/anterior femoral displacement. However, following a study by Sutton et al.,^24^ who investigated how natural knees responded to the inputs from the load control standard, this was reversed in the latest edition of the standard^25^ (published in 2014). AP translation is now defined as anterior tibia translation/posterior femoral displacement. The Leeds approach has consistently used anterior tibial translation in order to replicate femoral rollback when combined with setting the femoral centre on the distal radius. The polarity of the IE rotation input profile has also been reversed in the 2014 edition of the standard.^25^

Computational modelling is an attractive approach to wear simulation in total joint replacements, allowing parametric studies at substantially reduced cost and time.^26^ Computational wear models have been developed utilising either the simplified Archard’s wear law,^27^ based on the sliding distance and load,^26,28,29^ or the modified Archard’s wear law, based on the contact area and contact pressure.^30^ More recently, a new wear formula based on the contact area and sliding distance has been developed and applied to computational wear models at the University of Leeds.^4,6,31,32^ The computational wear predictions from this new wear formula have been validated against experimental studies.^5,31,32^

The aim of this study was to investigate the influence of simulation input conditions on the wear performance of a fixed-bearing TKR through a combined experimental and computational approach. The simulation input conditions investigated were input kinematic magnitudes, waveforms and directions of motion and position of the femoral CoR. Furthermore, studies were completed using conventional and moderately cross-linked polyethylene to determine whether the influence of these simulation input conditions varied with the material.

## Materials and methods

### Materials

The influence of simulation input conditions were investigated using a mid-size right Sigma fixed-bearing cruciate-retaining TKR (DePuy Synthes, Leeds, UK). These comprised Co-Cr-Mo alloy femoral components and polished Co-Cr-Mo tibial trays. In one test, Sigma CR150 femoral components were used (Table 2). The geometry of the CR150 has been optimised for high flexion and in the 0°–60° articulating region was equivalent to that of the standard Sigma geometry. The femoral components and tibial trays were used in multiple studies. However, their surface roughness was measured at the end of each study to confirm that there were no significant changes to the surfaces. Two types of polyethylene tibial inserts were investigated: (1) curved GUR1020 ultra-high-molecular-weight polyethylene (UHMWPE) inserts which had been sterilised in foil pouches by gamma irradiation (2.5–4 MRad) in a vacuum (Gamma Vacuum Foil (GVF^™^) material) and (2) curved moderately cross-linked GUR1020 UHMWPE (5 MRad irradiated and remelted) inserts (Moderately cross-linked (XLK^™^) material). Several sets of tibial inserts were used in these studies, where each set comprised of n = 6, further details of which can be found in Tables 1 and 2.

**Table 1.** Test order, duration and tibial insert set details for the GVF^™^ tibial inserts (n = 6 for each set).

CoR	Distal radius		ISO		
Kinematic condition	Intermediate kinematics	High kinematics	Modified intermediate kinematics	Modified high kinematics	ISO
Testing order	1	2	5	3	4
Tibial insert set	Set GVF 1^a^	Set GVF 2	Set GVF 3	Set GVF 3	Set GVF 3
Test duration	3 MC	5 MC	3 MC	3 MC	3 MC

CoR: centre of rotation; MC: million cycles; GVF: Gamma Vacuum Foil.

a Coupled with Sigma CR150 femoral.

**Table 2.** Test order, duration and tibial insert set details for the XLK^™^ tibial inserts (n = 6 for each set).

CoR	Distal radius		ISO		
Kinematic condition	Intermediate kinematics	High kinematics	Modified intermediate kinematics	Modified high kinematics	ISO
Testing order	1	2	4	3	N/A
Tibial insert set	Set XLK 1	Set XLK 1	Set XLK 2	Set XLK 2	N/A
Test duration	3 MC	3 MC	4 MC	3 MC	N/A

CoR: centre of rotation; MC: million cycles; XLK: moderately cross-linked.

### Experimental methodology

**Figure 1.**
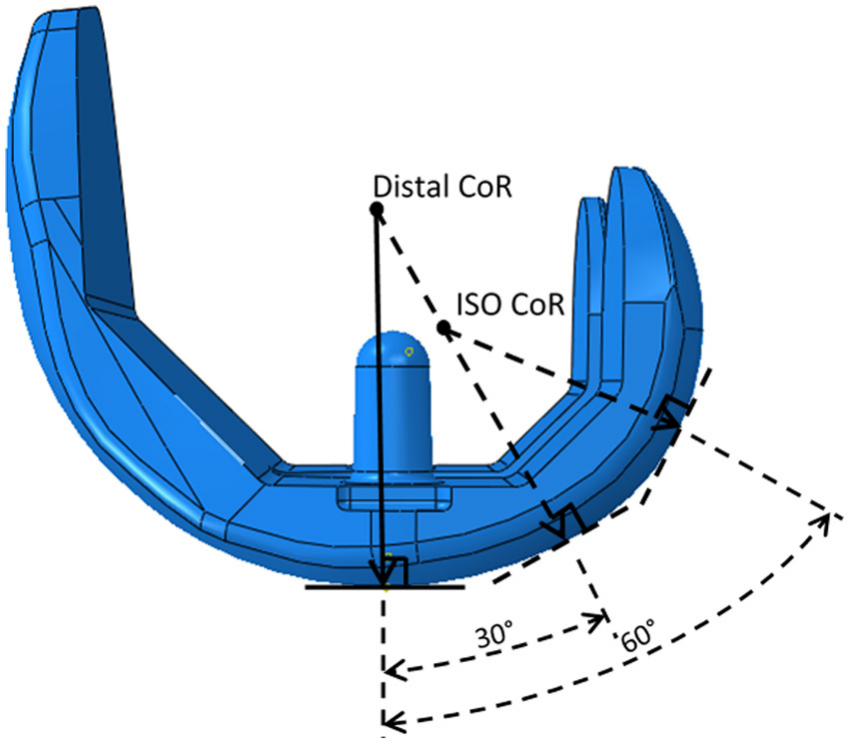
Resulting distal and ISO position of the femoral CoR on a Sigma CR femoral component.

The wear of the fixed-bearing knee replacements was investigated using a physiological six-station Leeds/Prosim knee wear simulator (Simulation Solutions, Stockport, UK), in which TKRs were mounted anatomically in each station. The central axis of each implant was offset from the aligned axes of applied load and tibial rotation from the centre of the joint by 7% of its width, in accordance with ISO 14243-1.^20^ The CoR of the femoral components was taken as either the distal radius of the implant, as indicated by the device design, or according to the ISO standard, which was an average of the femoral distal and posterior radii (Figure 1).^20^

**Figure 2.**
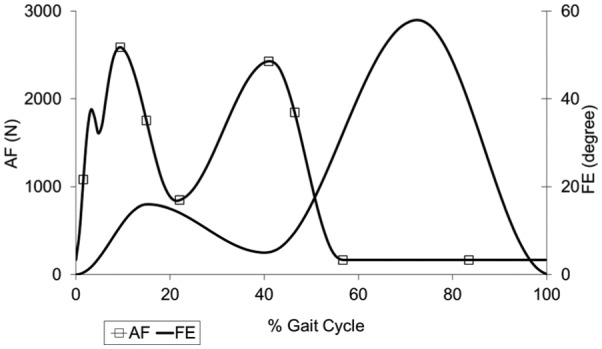
Axial force (AF) and flexion-extension (FE) input profiles.

**Figure 3.**
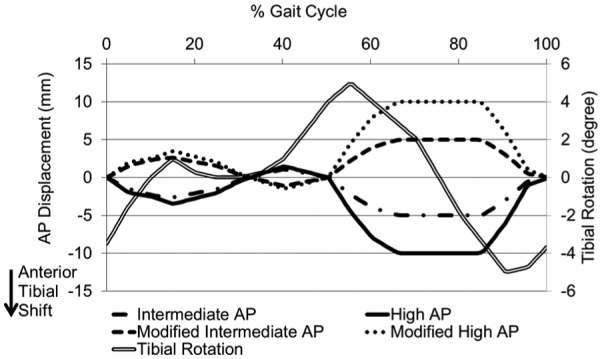
AP displacement and tibial rotation input profiles.

Each station had 6 degrees of freedom with four controlled axes of motion – axial load, femoral flexion, tibial IE rotation and tibial AP displacement. Abduction/adduction was allowed but not controlled. In order to eliminate station-specific differences, the samples were moved around the stations every million cycles (MC). Several test conditions were explored through the study (Table 3). The femoral axis loading (maximum 2600 N) and extension–flexion (0°–58°) input profiles were taken from the international standards^20,21^ for all test conditions and are shown in Figure 2. The IE tibial rotation was displacement controlled and set at ±5° based on the natural kinematics of the knee as described by Lafortune et al.^23^ and as shown in Figure 3. AP translation was displacement controlled for all studies, as this design of fixed-bearing knee replacement had minimal constraint and thus relies on soft tissue in vivo. The displacement test conditions used were ‘intermediate kinematics’ with an AP displacement of maximum 5 mm and ‘high kinematics’ with an AP displacement of maximum 10 mm. Consistent with previous studies,^6,7,14,19^ the distal-radius CoR was used with a polarity of anterior tibial shift (shown as negative AP motion in Figure 3) that produced femoral rollback. In studies using the ISO CoR position, it was necessary to reverse the direction of the AP displacement inputs in order to maintain the area of contact within the articulating region of the tibial insert. These kinematics were denoted ‘modified high’ and ‘modified intermediate’ kinematics for maximum positive displacements of 10 and 5 mm, respectively (Figure 3). The resulting posterior translation of the tibia prevented femoral rollback.

**Table 3.** Experimental test conditions.

CoR	Distal radius		ISO		
Kinematic condition	High kinematics	Intermediate kinematics	Modified high kinematics	Modified intermediate kinematics	ISO
Maximum AP displacement and polarity	−10-mm anterior tibial shift	−5-mm anterior tibial shift	10-mm posterior tibial shift	5-mm posterior tibial shift	5-mm posterior tibial shift
Internal–external rotation	±5°	±5°	±5°	±5°	−2° to +5.5°

CoR: centre of rotation; AP: anterior–posterior.

**Figure 4.**
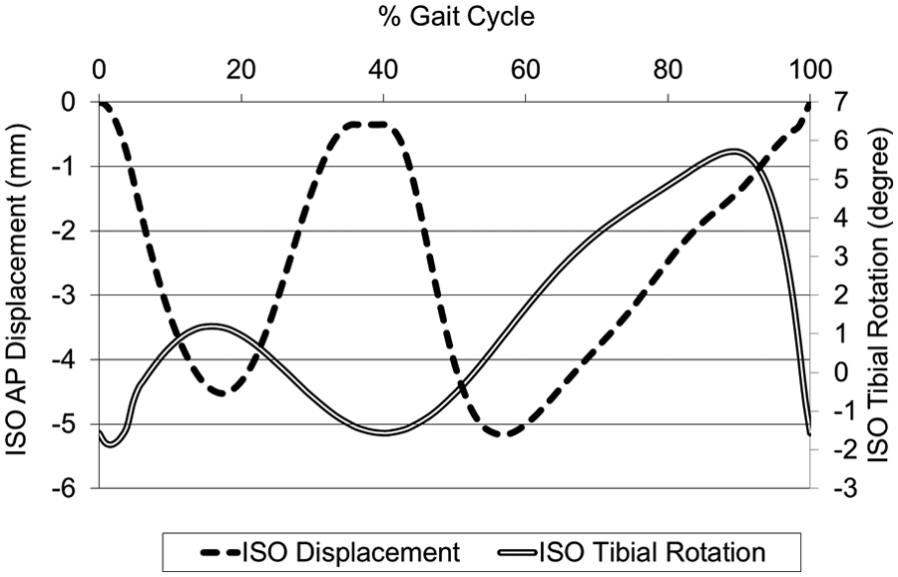
ISO AP displacement and tibial rotation input profiles.

An additional comparative study using the full ISO conditions taken from the 2004 version of the displacement control standard^21^ (both CoR position and displacement profiles as shown in Figure 4) was conducted upon the conventional polyethylene (GVF) inserts. This was performed to further investigate the influence of simulation input conditions on the contact area, cross-shear and hence wear; specifically, the reduced amplitude and differing shape of the tibial rotation input profile compared to the Leeds profiles and the shape of the AP displacement profile.

All studies were conducted for a minimum period of 3 MC for each condition (Tables 1 and 2). The simulator was run at a frequency of 1 Hz. The lubricant used was 25% new-born calf serum supplemented with 0.03% (v/v) sodium azide to retard bacterial growth and was changed every 0.33 MC. Prior to testing, all inserts were soaked in deionised water for a minimum period of 4 weeks. This allowed an equilibrated fluid absorption level to be achieved prior to the commencement of the wear study reducing variability due to fluid weight gain. Wear of the tibial inserts was determined gravimetrically using a Mettler AT201 (Mettler-Toledo, Leicester, UK) digital microbalance, which had a readability of 0.01 mg. The volumetric wear was calculated from the weight loss measurements using a density of 0.93 mg/mm^3^ for both polyethylene materials and using unloaded soak controls to compensate for moisture uptake.

Statistical analysis of the data was performed by first testing for homogeneity of variances and the means compared using the one-way analysis of variance (ANOVA) with 95% confidence interval. A post-hoc test was subsequently performed as appropriate using the Tukey’s test and significance was taken at p < 0.05. This statistical analysis was performed using SPSS statistics (IBM SPSS, ver. 22).

### Computational methodology

The computational framework corresponded directly with the experimental simulation. The computational model was further used to decouple the effect of kinematic polarity and the position of the femoral CoR on the wear prediction. The computational wear model for the knee implants was based on the contact area (*A*), sliding distance (*S*) and an independent experimentally determined non-dimensional wear coefficient (*C*) to determine the wear volume (*W*). *C* was a function of cross-shear ratio (*CSR*). *W* was calculated according to the following equation^31^

(1) W=C×A×S

The *CSR* was defined based on the unified theory of wear and frictional work by Wang^33^ and the work by Kang et al.^34^ to account for the strain hardening in the articulating polyethylene surface. The relationships between the non-dimensional wear coefficient and *CSR* were determined from independent experimental pin-on-plate wear studies by Kang et al.^34^ and Abdelgaied et al.^35^ for conventional and moderately cross-linked UHMWPE materials, respectively. These independent experimental pin-on-plate studies determined the material wear coefficient as an input to the model.

Conventional and moderately cross-linked UHMWPE materials were modelled in ABAQUS (ABAQUS 6.12-2) using stress–strain data supplied by the manufacturer (DePuy International, UK) as isotropic elastic–plastic^36^ and isotropic elastic materials, respectively. The modulus of elasticity and Poisson’s ratio were taken as 463 MPa, 0.46^37^ and 673 MPa, 0.46^5^ for conventional and moderately cross-linked UHMWPE, respectively. A mesh sensitivity study resulted in a total number of elements of 42,777 for the tibial insert using modified quadratic tetrahedral elements (C3D10M). The femoral component was modelled as a rigid body.^38^ Isotropic penalty contact was used to define the surface-to-surface contact between the tibial and femoral contact surfaces with a coefficient of friction of 0.04.^36^ The computational model was run for 3 MC for each condition. The output sliding distances and contact areas were used to calculate the *CSR* and therefore the volumetric wear rate in mm^3^/MC. The computational framework is described more extensively elsewhere.^31^

## Results

**Figure 5.**
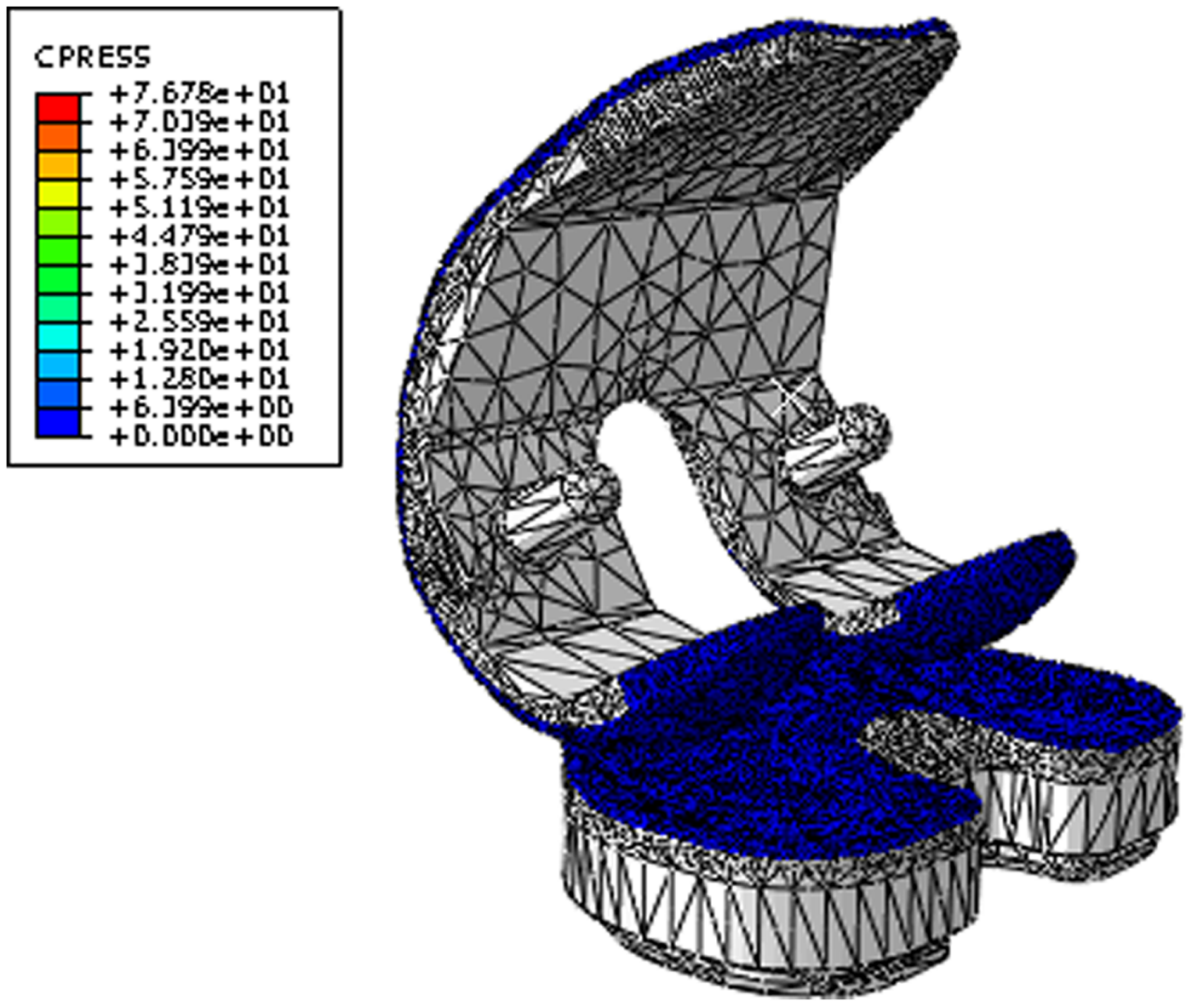
Contact stress and location for the Sigma fixed-bearing knee replacement under Leeds high kinematic inputs and ISO CoR position.

Preliminary experimental studies using the ISO femoral CoR position and standard Leeds high kinematic test conditions (AP anterior tibial shift of 10 mm) resulted in the contact between the femoral bearing and insert moving outside the articulating region of the tibial insert. This effect was also demonstrated through the computational contact modelling (Figure 5), and hence, the direction of motion for the AP input kinematics for the ISO position of the femoral CoR was reversed in order to maintain contact within the articulating region of the insert.

**Figure 6.**
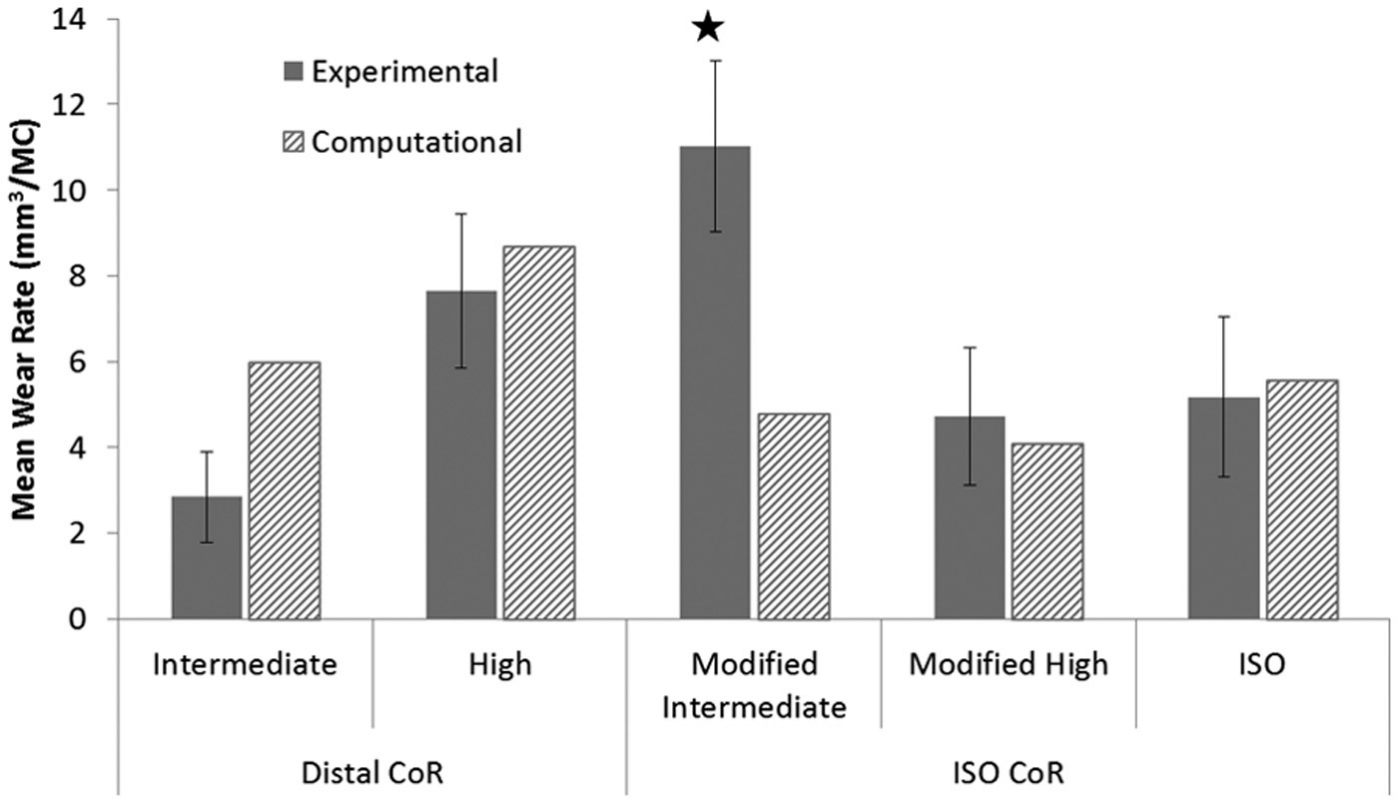
Experimental and computational mean wear rates for fixed-bearing total knee replacements with GVF-bearing material under different conditions (95% confidence limits indicated for experimental studies). ^*^Higher wear rate potentially due to damage of the contact surface and sub-surface cracking.

**Figure 7.**
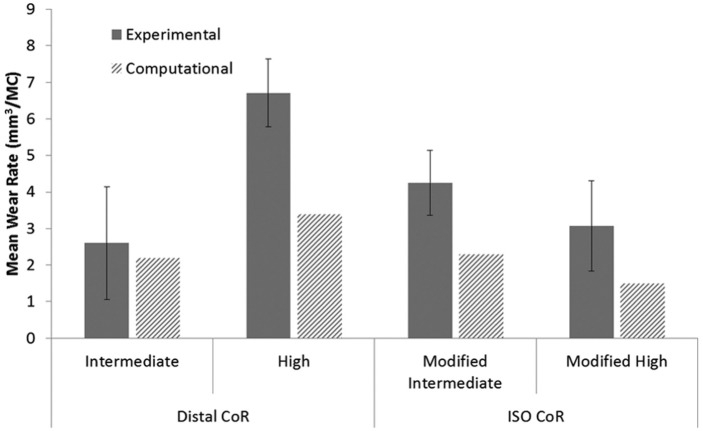
Experimental and computational mean wear rates for fixed-bearing total knee replacements with XLK-bearing material under different conditions (95% confidence limits indicated for experimental studies).

The position of the femoral CoR and the input kinematics were shown to have a significant influence on the experimental wear rates of the GVF inserts (Figure 6) (p < 0.05 ANOVA). The mean wear rate with 95% confidence limits on the distal CoR with high kinematics, with AP motion polarity producing rollback (7.6 ± 1.8 mm^3^/MC) was significantly higher than the ISO CoR position with modified high kinematics (4.7 ± 1.6 mm^3^/MC; ANOVA, p < 0.05). In addition, increasing the kinematic level through doubling the magnitude of the AP displacement on distal CoR from intermediate to high kinematics increased the wear rate significantly from 2.9 ± 1.1 to 7.6 ± 1.8 mm^3^/MC, respectively (ANOVA, p < 0.01). In contrast, increasing the kinematic level on the ISO CoR from intermediate to high kinematics significantly decreased the wear rate from 11.0 ± 1.9 to 4.7 ± 1.6 mm^3^/MC, respectively (ANOVA, p < 0.01). However, this result should be treated with caution, as the experimentally measured high wear rate for the ISO position of the femoral CoR and modified intermediate kinematics was partly attributed to damage of the contact surface and sub-surface cracking of the GVF inserts. This was suspected to have occurred due to oxidative degradation in line with the age of the inserts (Figure 6). There was no significant difference between the modified high and ISO kinematic conditions under the ISO CoR position (ANOVA, p > 0.05), with a wear rate of 5.2 ± 1.9 mm^3^/MC observed under ISO input kinematics. The moderately cross-linked XLK inserts showed similar wear rate trends to those of the conventional GVF material (Figure 7). The XLK experimentally measured wear rates under different kinematic conditions and position of the femoral CoR conditions were, however, lower than those of the GVF material for all conditions (Figures 6 and 7).

**Figure 8.**
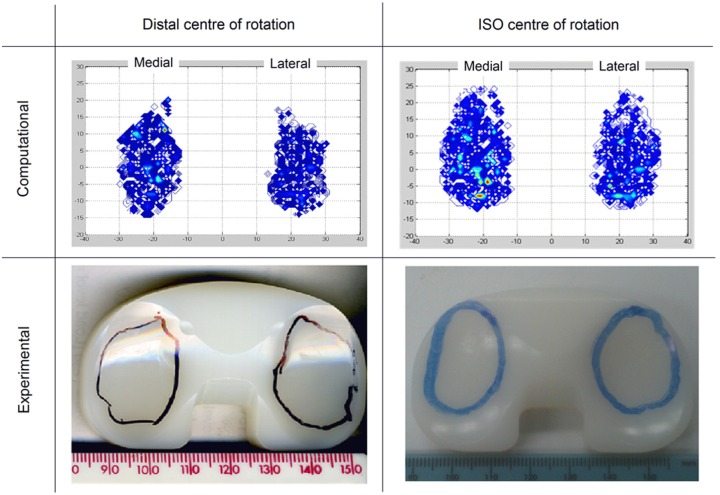
Comparison of wear scars at completion of study for distal and ISO CoRs after high and modified high kinematics, respectively.

The mean wear scars at the completion of experimental simulation under distal/high and ISO/modified high kinematics were compared qualitatively (Figure 8). The experimental and the computational studies showed that the wear scars under the ISO/modified high kinematic conditions were located more anteriorly than the distal/high scars.

**Figure 9.**
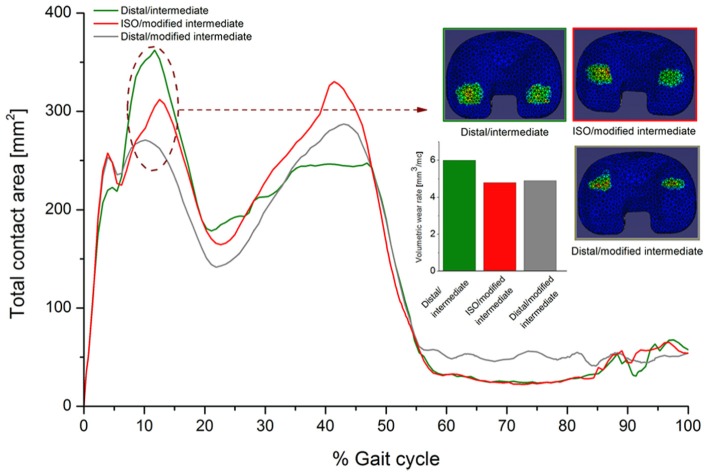
Effect of kinematic polarity and position of the femoral CoR on the predicted wear rate, cross-shear ratio and contact area throughout the gait cycle under intermediate kinematic inputs for the GVF material.

Although the computational predicted absolute wear rate values often led outside of the 95% confidence limits from the experimental tests, in terms of wear rate trends and qualitative observations of the wear scars, there was generally good agreement between the experimental and computational wear results for all conditions and materials (Figures 6–8). The computational model was further used to decouple the effect of kinematic polarity and position of the femoral CoR on wear prediction. This was performed through comparison of the conditions of distal CoR/intermediate conditions and distal CoR/modified intermediate conditions to determine the influence of the direction of AP motion, and through comparison of distal CoR/modified intermediate conditions and ISO CoR/modified intermediate conditions to determine the influence of the CoR (Figure 9). It was not possible to run either the experimental or computational simulation under modified high and high kinematic inputs for the distal and ISO CoRs, respectively, due to the femoral component rolling off the tibial insert. Hence, the effect of kinematic polarity and position of the femoral CoR were examined computationally under the lower AP translation intermediate kinematic conditions. Reversing the intermediate kinematic inputs polarity under the distal CoR reduced the predicted wear rate from 6 to 4.8 mm^3^/MC. Changing the position of the femoral CoR from ISO position to distal (under modified intermediate kinematic inputs polarity, that is, posterior tibial shift) did not, however, affect the predicted wear rate (4.9 mm^3^/MC under ISO femoral CoR compared to 4.8 mm^3^/MC under distal femoral CoR).

**Figure 10.**
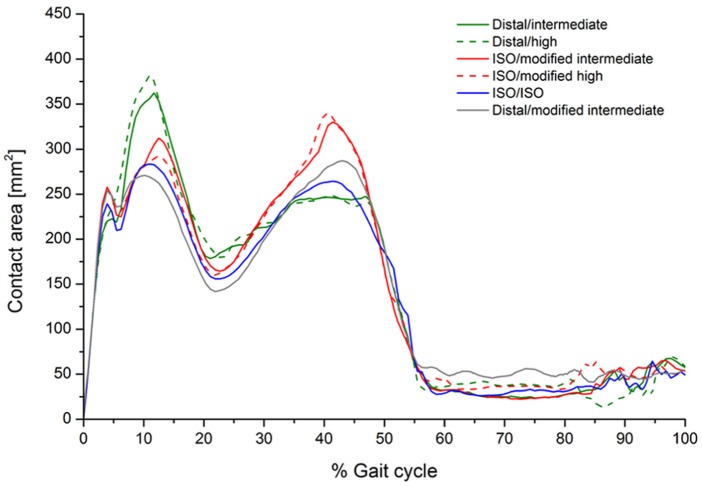
Predicted contact area throughout the gait cycle for GVF material.

The computationally predicted contact area throughout the gait cycle for the GVF inserts for all test conditions is summarised in Figure 10. In addition, the computationally predicted average wear rates and *CSR* for GVF inserts for all test conditions are summarised in Table 4.

**Table 4.** Computationally predicted average wear rates and cross-shear ratios for GVF inserts.

Test conditions (CoR/kinematics)	Predicted average wear rate (mm^3^/MC)	Predicted average cross-shear ratio
Distal/intermediate	6.0	0.0253
Distal/high	8.7	0.0505
ISO/modified intermediate	4.9	0.0057
ISO/modified high	4.1	0.0041
Distal/modified intermediate	4.8	0.0017
ISO/ISO	5.6	0.0072

## Discussion

This is the first study investigating the influence of knee simulation input conditions comprising the position of the femoral CoR, input kinematics and direction of motion on the wear of a fixed-bearing TKR using a combined experimental and computational simulation approach. The experimental and computational studies have both demonstrated the effects the change in the CoR position and kinematic inputs, and most importantly the direction of the AP translation, had on the relative contact points between the femoral and tibial components and hence wear during a standard gait cycle (Figures 8–10). In order to maintain contact within the articulating region of the insert, when the position of the femoral CoR was changed from the distal to the ISO CoR, the direction of the standard Leeds AP displacement required reversal. The change in the wear rate was therefore due to the combined effect of changing the position of the femoral CoR and the direction of AP motion. The computational simulation was used to decouple this effect and provide further information on contact area, contact location and relative motion at the articulating surfaces.

Specifically, the experimental wear simulation showed that changing the kinematic inputs from intermediate to high kinematics, with maximum amplitudes of 5- and 10-mm displacement in the AP direction, respectively, significantly increased the mean wear rate under the distal CoR for both GVF and XLK insert materials. This increase in the wear rate with increase in the AP displacement was, however, not observed when the ISO CoR was used. Indeed, a reduction in the wear rate of 60% and 30% for GVF and XLK insert materials, respectively, was observed (Figures 6 and 7). This was confounded further by the requirement to reverse the direction of AP motion in order to maintain the contact within the articulating area.

On the distal CoR, where the polarity of AP motion replicates rollback and moves the contact point posteriorly (Figures 8 and 10), there was higher conformity between the articulating surfaces and therefore higher *CRSs*. Under high kinematics, the contact extended posteriorly with an average *CSR* of 0.0505. While for intermediate kinematics, the contact extended less posteriorly with an average *CSR* of 0.0253 and hence resulted in lower wear. In contrast, on the ISO CoR with posterior tibial shift, the AP translation moved the contact point anteriorly with lower conformity between the articulating surfaces and therefore lower *CSRs*. This had the effect of reducing average *CSR* and wear when high kinematics and high AP motion of maximum 10 mm was applied (*CSR* = 0.0041), but resulted in a higher average *CSR* and wear when intermediate AP of 5 mm was used (*CSR* = 0.0057). The *CSR* was not the only factor which controlled the wear performance. However, for the same CoR, the change in the contact area with the change in kinematic input was not significant, and therefore, the effect of *CSR* on wear was the dominant factor.

On the ISO CoR, changing the kinematics from a maximum 10-mm AP tibial shift and ±5° tibial rotation to ISO inputs of 5-mm posterior tibial shift and −2°/+6° tibial rotation produced similar wear rates for GVF inserts (Figure 6). The computational prediction for the *CSR* between these conditions differed by a factor of 2, with the ISO conditions having the higher *CSR*. The total contact area was, however, lower than that predicted under the condition of maximum 10-mm AP tibial shift and ±5° tibial rotation, and the net effect was therefore not significantly different.

The 2004 version of the ISO displacement control standard^21^ for knee wear simulation maintained the contact in the centre of the tibia and prevented femoral rollback, producing a lower cross-shear, lower wearing simulation, which will not replicate the range of kinematic conditions and wear rates found clinically. A recently revised version of the ISO displacement control standard^25^ has reversed the direction of the AP motion, with an anterior tibial shift of maximum 5 mm. This brings the AP polarity in line with the Leeds waveforms, although the CoRs are still different. The polarity of the tibial rotation profile has also been reversed in the 2014 version of the standard compared to the previous. However, there may still be a need to consider the position of the femoral CoR to match the physiological set-up and replicate clinical motion (femoral rollback).

The dependence of wear on simulation input conditions as demonstrated in this study suggests that these conditions should be carefully controlled when comparing the wear of different designs or materials. This appropriate level of control can be achieved through the use of displacement control simulation. Simulation using force control is associated with more variation in the delivered kinematics to the TKRs under test and hence more variation in wear, masking the differences between different designs and/or materials. Therefore, in order to determine wear when variables such as design or material are being studied, the variation in kinematics should be carefully controlled and the displacement control regime is a more appropriate way to achieve this.

Similar trends were shown for the moderately cross-linked material, although lower wear rates were found overall due to the higher level of cross-linking (Figure 7) in line with other studies of cross-linked tibial inserts.^7,39^

It is important to note that this investigation into the influence of simulation input conditions was performed using a single design of fixed-bearing TKR, and the influence of these conditions may differ with different knee replacement designs. Further limitations to the study include a sample size of six, which may reduce the effectiveness and power of the study. However, n = 6 was chosen as the number of replicates and power, because it had been used in previous studies in knee simulators and had been able to differentiate difference in wear rates between different kinematic conditions and different materials,^4,6,7,10,14,17–19^ with sample sizes of three being used by other groups.^22,39^ For cross-linked polyethylene where lower absolute wear rates are observed, the difference in absolute terms between different conditions becomes lower, and future work may need to consider increasing the number of replicates in the study, when investigating wear rates of less than 5 mm^3^. However, the clinical relevance of these small absolute differences in the lower wear rates becomes less. In several of the tests, the same components were used, meaning that the results may not have been strictly independent; it is possible that changes in the surface topography of the implants as a result of one test may have influenced the wear of another where the same set of components were used. However, their surface roughness was routinely measured at the end of each study to confirm that there were no significant changes to the surfaces and mitigate this risk.

In terms of limitations of the computational model, the experimental wear coefficients used as an input to the computational model were calculated from wear data obtained from multi-directional pin-on-plate studies under constant loading conditions and against smooth counterfaces. Dynamic loading in a pin-on-plate wear test can increase the wear volume^40^ and hence the wear coefficient.^32^ Additionally, it can be difficult to obtain such smooth surface profiles on cobalt chrome femoral surfaces. Moreover, the model is only valid under specified loading conditions. For these specified conditions, the surface wear mechanism dominates. Other test conditions may be associated with higher contact pressures, meaning different wear mechanisms may therefore take place.^5^

## Conclusion

The most important factor influencing the wear of the fixed-bearing knee replacement investigated in this study was the position of the relative contact point at the femoral component and tibial insert interface. This was dependent on a combination of many parameters including input displacement magnitudes, waveforms, direction of motion and femoral CoR. The influence of the simulation input conditions did not vary with the two materials investigated in this study, with similar trends observed for both conventional and moderately cross-linked bearing materials. This study provides further evidence and supports that in order to study variables such as design and material in TKR, it is important to carefully control knee simulation conditions. This can be more effectively achieved through the use of displacement control simulation.

## References

[1] The NJR Editorial Board. 12th annual report of the National Joint Registry for England, Wales, Northern Ireland and the Isle of Man, http://www.njrcentre.org.uk/njrcentre/Portals/0/Documents/England/Reports/12th%20annual%20report/NJR%20Online%20Annual%20Report%202015.pdf

[2] Dalury DF Gonzales RA Adams MJ Midterm results with the PFC sigma total knee arthroplasty system. J Arthroplasty 2008; 23: 175–181. 18280409 10.1016/j.arth.2007.03.039

[3] Hooper G Rothwell A Frampton C. The low contact stress mobile-bearing total knee replacement: a prospective study with a minimum follow-up of ten years. J Bone Joint Surg Br 2009; 91: 58–63. 19092005 10.1302/0301-620X.91B1.20484

[4] Fisher J Jennings L Galvin A 2009 knee society presidential guest lecture: polyethylene wear in total knees. Clin Orthop Relat Res 2010; 468: 12–18. 19669846 10.1007/s11999-009-1033-1 PMC2795814

[5] Abdelgaied A Brockett CL Liu F The effect of insert conformity and material on total knee replacement wear. Proc IMechE, Part H: J Engineering in Medicine 2014; 228: 98–106. 10.1177/0954411913513251 PMC4361477 24297773

[6] Galvin AL Kang L Udofia I Effect of conformity and contact stress on wear in fixed-bearing total knee prostheses. J Biomech 2009; 42: 1898–1902. 19524245 10.1016/j.jbiomech.2009.05.010

[7] Brockett CL Jennings LM Hardaker C Wear of moderately cross-linked polyethylene in fixed bearing total knee replacement. Proc IMechE, Part H: J Engineering in Medicine 2012; 226: 529–535. 10.1177/0954411912445265 22913100

[8] Utzschneider S Paulus A Datz JC Influence of design and bearing material on polyethylene wear particle generation in total knee replacement. Acta Biomater 2009; 5: 2495–2502. 19375997 10.1016/j.actbio.2009.03.016

[9] Abdelgaied A Brockett C Liu F Effect of backside wear and bearing material on wear performance of rotating platform mobile bearings. Bone Joint J Orthop Proc Suppl 2013; 95: 214.

[10] McEwen HMJ Barnett PI Bell CJ The influence of design, materials and kinematics on the in vitro wear of total knee replacements. J Biomech 2005; 38: 357–365. 15598464 10.1016/j.jbiomech.2004.02.015

[11] Affatato S Bracco P Sudanese A. In vitro wear assessments of fixed and mobile UHMWPE total knee replacement. Mater Design 2013; 48: 44–51.

[12] Affatato S Grillini L Battaglia S Does knee implant size affect wear variability? Tribol Int 2013; 66: 174–181.

[13] Affatato S Leardini W Rocchi M Investigation on wear of knee prostheses under fixed kinematic conditions. Artif Organs 2008; 32: 13–18. 18181798 10.1111/j.1525-1594.2007.00455.x

[14] Jennings L Bell C Ingham E The influence of femoral condylar lift-off on the wear of artificial knee joints. Proc IMechE, Part H: J Engineering in Medicine 2007; 221: 305–314. 10.1243/09544119JEIM215 17539585

[15] DesJardins J Rusly R. Single flexion-axis selection influences femoral component alignment and kinematics during knee simulation. Proc IMechE, Part H: J Engineering in Medicine 2011; 225: 762–768. 10.1177/0954411911400534 21922953

[16] Zietz C Reinders J Schwiesau J Experimental testing of total knee replacements with UHMW-PE inserts: impact of severe wear test conditions. J Mater Sci Mater Med 2015; 26: 134. 25716024 10.1007/s10856-015-5470-y

[17] McEwen HMJ Fisher J Goldsmith AAJ Wear of fixed bearing and rotating platform mobile knees subjected to high levels of internal and external tibial rotation. J Mater Sci Mater Med 2001; 12: 1049–1052. 15348363 10.1023/a:1012850224565

[18] Barnett PI McEwen HMJ Auger DD Investigation of wear of knee prostheses in a new displacement/force-controlled simulator. Proc IMechE, Part H: J Engineering in Medicine 2002; 216: 51–61. 10.1243/0954411021536289 11908483

[19] Barnett PI Fisher J Auger DD Comparison of wear in a total knee replacement under different kinematic conditions. J Mater Sci Mater Med 2001; 12: 1039–1042. 15348361 10.1023/a:1012894023657

[20] ISO14243-1:2009. Implants for surgery – wear of total knee-joint prostheses – part 1: loading and displacement parameters for wear-testing machines with load control and corresponding environmental conditions for test.

[21] ISO14243-3:2004. Implants for surgery – wear of total knee-joint prostheses – part 3: loading and displacement parameters for wear-testing machines with displacement control and corresponding environmental conditions for test.

[22] Johnson TS Laurent MP Yao JQ The effect of displacement control input parameters on tibiofemoral prosthetic knee wear. Wear 2001; 250: 222–226.

[23] Lafortune MA Cavanagh PR Sommer Iii HJ Three-dimensional kinematics of the human knee during walking. J Biomech 1992; 25: 347–357. 1583014 10.1016/0021-9290(92)90254-x

[24] Sutton LG Werner FW Haider H In vitro response of the natural cadaver knee to the loading profiles specified in a standard for knee implant wear testing. J Biomech 2010; 43: 2203–2207. 20451913 10.1016/j.jbiomech.2010.03.042

[25] ISO14243-3:2014. Implants for surgery – wear of total knee-joint prostheses – part 3: loading and displacement parameters for wear-testing machines with displacement control and corresponding environmental conditions for test.

[26] Knight LA Pal S Coleman JC Comparison of long-term numerical and experimental total knee replacement wear during simulated gait loading. J Biomech 2007; 40: 1550–1558. 17084405 10.1016/j.jbiomech.2006.07.027

[27] Archard JF Hirst W. The wear of metals under unlubricated conditions. P Roy Soc Lond A Mat 1956; 236: 397–410.

[28] Barbour PSM Barton DC Fisher J. The influence of contact stress on the wear of UHMWPE for total replacement hip prostheses. Wear 1995; 181–183: 250–257.

[29] Maxian TA Brown TD Pedersen DR A sliding-distance-coupled finite element formulation for polyethylene wear in total hip arthroplasty. J Biomech 1996; 29: 687–692. 8707799 10.1016/0021-9290(95)00125-5

[30] Willing R Kim IY. Three dimensional shape optimization of total knee replacements for reduced wear. Struct Multidiscip O 2009; 38: 405–414.

[31] Abdelgaied A Liu F Brockett C Computational wear prediction of artificial knee joints based on a new wear law and formulation. J Biomech 2011; 44: 1108–1116. 21329928 10.1016/j.jbiomech.2011.01.027

[32] Liu F Galvin A Jin Z A new formulation for the prediction of polyethylene wear in artificial hip joints. Proc IMechE, Part H: J Engineering in Medicine 2010; 225: 16–24. 10.1243/09544119JEIM819 21381484

[33] Wang A. A unified theory of wear for ultra-high molecular weight polyethylene in multi-directional sliding. Wear 2001; 248: 38–47.

[34] Kang L Galvin AL Brown TD Quantification of the effect of cross-shear on the wear of conventional and highly cross-linked UHMWPE. J Biomech 2008; 41: 340–346. 17936763 10.1016/j.jbiomech.2007.09.005 PMC2239346

[35] Abdelgaied A Brockett CL Liu F Quantification of the effect of cross-shear and applied nominal contact pressure on the wear of moderately cross-linked polyethylene. Proc IMechE, Part H: J Engineering in Medicine 2013; 227: 18–26. 10.1177/0954411912459423 23516952

[36] Godest AC Beaugonin M Haug E Simulation of a knee joint replacement during a gait cycle using explicit finite element analysis. J Biomech 2002; 35: 267–275. 11784545 10.1016/s0021-9290(01)00179-8

[37] Bartel D Rawlinson J Burstein A Stresses in polyethylene components of contemporary total knee replacements. Clin Orthop Relat Res 1995; 317: 76–82. 7671500

[38] Carr BC Goswami T. Knee implants – review of models and biomechanics. Mater Design 2009; 30: 398–413.

[39] Utzschneider S Harrasser N Schroeder C Wear of contemporary total knee replacements – a knee simulator study of six current designs. Clin Biomech 2009; 24: 583–588. 10.1016/j.clinbiomech.2009.04.007 19450910

[40] Barbour PSM Barton DC Fisher J. The influence of stress conditions on the wear of UHMWPE for total joint replacements. J Mater Sci Mater Med 1997; 8: 603–611. 15348829 10.1023/a:1018515318630

